# Improved classification accuracy in 1- and 2-dimensional NMR metabolomics data using the variance stabilising generalised logarithm transformation

**DOI:** 10.1186/1471-2105-8-234

**Published:** 2007-07-02

**Authors:** Helen M Parsons, Christian Ludwig, Ulrich L Günther, Mark R Viant

**Affiliations:** 1 Centre for Systems Biology, The University of Birmingham, Edgbaston, Birmingham, B15 2TT, UK; 2 The Henry Wellcome Building for Biomolecular NMR Spectroscopy, The University of Birmingham, Edgbaston, Birmingham, B15 2TT, UK; 3 School of Biosciences, The University of Birmingham, Edgbaston, Birmingham, B15 2TT, UK

## Abstract

**Background:**

Classifying nuclear magnetic resonance (NMR) spectra is a crucial step in many metabolomics experiments. Since several multivariate classification techniques depend upon the variance of the data, it is important to first minimise any contribution from unwanted technical variance arising from sample preparation and analytical measurements, and thereby maximise any contribution from wanted biological variance between different classes. The generalised logarithm (glog) transform was developed to stabilise the variance in DNA microarray datasets, but has rarely been applied to metabolomics data. In particular, it has not been rigorously evaluated against other scaling techniques used in metabolomics, nor tested on all forms of NMR spectra including 1-dimensional (1D) ^1^H, projections of 2D ^1^H, ^1^H J-resolved (pJRES), and intact 2D J-resolved (JRES).

**Results:**

Here, the effects of the glog transform are compared against two commonly used variance stabilising techniques, autoscaling and Pareto scaling, as well as unscaled data. The four methods are evaluated in terms of the effects on the variance of NMR metabolomics data and on the classification accuracy following multivariate analysis, the latter achieved using principal component analysis followed by linear discriminant analysis. For two of three datasets analysed, classification accuracies were highest following glog transformation: 100% accuracy for discriminating 1D NMR spectra of hypoxic and normoxic invertebrate muscle, and 100% accuracy for discriminating 2D JRES spectra of fish livers sampled from two rivers. For the third dataset, pJRES spectra of urine from two breeds of dog, the glog transform and autoscaling achieved equal highest accuracies. Additionally we extended the glog algorithm to effectively suppress noise, which proved critical for the analysis of 2D JRES spectra.

**Conclusion:**

We have demonstrated that the glog and extended glog transforms stabilise the technical variance in NMR metabolomics datasets. This significantly improves the discrimination between sample classes and has resulted in higher classification accuracies compared to unscaled, autoscaled or Pareto scaled data. Additionally we have confirmed the broad applicability of the glog approach using three disparate datasets from different biological samples using 1D NMR spectra, 1D projections of 2D JRES spectra, and intact 2D JRES spectra.

## Background

Metabolomics relies extensively upon the multivariate analysis of data [1]. Unsupervised data mining tools such as principal component analysis (PCA) [2,3] and hierarchical clustering [4,5] and supervised methods such as partial least squares discriminant analysis (PLS-DA) [1,6] are commonly used to search for patterns and other features within metabolomic data sets. Many multivariate techniques evaluate possible relationships between samples by examining the variance of the data [3,7], the most notable being PCA for which principal components (PCs) are calculated along the directions of maximum variance. Hence the structure of the variance within a metabolomics data set can have a major effect on the output of the multivariate analyses. It is therefore important to assess (and modify appropriately) the variance structure of a metabolomics data set prior to multivariate analysis. Variation between samples can be broadly classified into one of two types – 'technical' and 'biological' [2,8]. Technical variance is created by the experimental procedure, and includes sample preparation and analytical measurement errors, whilst biological variance is the inherent variation between samples created by genetic differences, pathological or environmental factors, etc [8]. Clearly, the technical variance does not contribute any useful information to discriminate between different biological sample classes and so, ideally, this variance would not contribute to any multivariate analyses.

Data processing methods can be used to affect the structure of the variance of experimental data sets, helping to focus the subsequent multivariate analysis onto more biologically relevant information arising from the biological variance [2,9]. Common processing methods in nuclear magnetic resonance (NMR) spectroscopy based metabolomics include autoscaling [1,2] and Pareto scaling [1,2,5,10]. The generalised logarithm (glog) has also been investigated, but only in a limited number of studies [11]. For each variable in the spectrum, the glog transforms the intensity at that point to a value dependent on both the original intensity and the value of a transform parameter. The equation for the glog transform is shown below, where y represents the untransformed data, λ is the transform parameter, and z is the transformed data.

z=ln⁡(y+y2+λ)
 MathType@MTEF@5@5@+=feaafiart1ev1aaatCvAUfKttLearuWrP9MDH5MBPbIqV92AaeXatLxBI9gBaebbnrfifHhDYfgasaacH8akY=wiFfYdH8Gipec8Eeeu0xXdbba9frFj0=OqFfea0dXdd9vqai=hGuQ8kuc9pgc9s8qqaq=dirpe0xb9q8qiLsFr0=vr0=vr0dc8meaabaqaciaacaGaaeqabaqabeGadaaakeaacqWG6bGEcqGH9aqpcyGGSbaBcqGGUbGBcqGGOaakcqWG5bqEcqGHRaWkdaGcaaqaaiabdMha5naaCaaaleqabaGaeGOmaidaaOGaey4kaSccciGae83UdWgaleqaaOGaeiykaKcaaa@3B6A@

Autoscaling is a processing technique in which the variance of each variable is scaled to unity and the mean of each variable is set to zero [5]. In Pareto scaling each variable's intensity is scaled by the square root of the standard deviation of that variable [3], producing a data set where the variance changes from variable to variable, but the range of variance across each spectrum is much reduced from the initial, unscaled data.

The glog is a transformation that was originally applied to microarray data [12,13] and is based on the two-component error model [14]. Specifically, the measurement error of an observation is characterised by one component representing the error of the data as being proportional to the intensity of the measurement, and a second, additive, component of the error characterising the noise. Previously, the glog transform has been applied to one-dimensional (1D) NMR data as first shown by Purohit et al [11]. Unlike autoscaling and Pareto scaling, the glog transform initially requires a single parameter to be calibrated from a series of 'technical replicates'. These replicates must be recorded from one biological sample that has been divided into five or more components, each of which is subject to independent sample preparation and NMR analysis. The variance within this data set arises solely from technical sources [2,8], upon which the glog transform is calibrated [11]. Hence, when the glog is then applied to a biological data set it effectively reduces the amount of technical variance present, leaving the biological variance to dominate any subsequent multivariate analysis. To date, the glog transform has not been compared against other processing techniques used in metabolomics. Furthermore, the calibrated glog transform has only been tested using a single, relatively small 1D NMR data set [11]. Recently, due largely to severe peak congestion in 1D NMR spectra, there has been a significant increase in the range of 2D NMR experiments conducted in metabolomics [15-21]. Although many of these experiments require substantially longer acquisition times and so are not appropriate for high throughput metabolomics, 2D J-resolved (JRES) spectroscopy has been shown to provide spectra with low peak congestion and high metabolite specificity in a short acquisition time [15,20]. Consequently, several multivariate analyses of 1D projections of 2D JRES spectra have been reported [15,18-21]. To our knowledge the applicability of the glog transform, including the initial calibration of the function using technical replicates, has not been evaluated for these 1D projections of 2D JRES spectra, nor for the analysis of the intact 2D JRES spectra.

Here, we first aimed to evaluate comprehensively the glog transform compared to two other commonly used scaling methods in NMR metabolomics as well as against unscaled data. This evaluation was conducted using three disparate data sets to confirm the broad applicability of the approach, including: urine samples to discriminate between two dog breeds, muscle tissue extracts to discriminate between hypoxia and normoxia in marine mussels, and liver tissue extracts to discriminate between fish collected from two different rivers. The performances of each of the scaling methods – autoscaling, Pareto and glog – were assessed by conducting PCA of each of the processed two-class data sets. This was achieved by calculating the sensitivities and specificities derived from applying linear discriminant analysis (LDA) to each of the resulting PCA scores plots. The effect of each scaling method upon the ability to discover potential metabolic biomarkers was also investigated. This was accomplished by selecting the largest peaks in the PCA loadings plots and then evaluating if the corresponding peaks in the NMR spectra were of significantly different intensity between the biological classes. Secondly, we aimed to evaluate the applicability of the glog transform for 1D NMR spectra, 1D projections of 2D JRES spectra, and intact 2D JRES spectra. This enabled the first NMR metabolomics study of intact 2D JRES spectra; including the reconstruction of the PCA loadings plot to a 2D format analogous to the JRES spectra, which is anticipated to have significant benefit in terms of the ease of metabolite identification. During this second aim we also sought to extend the glog transform to reduce the deleterious effects of noise.

## Results and Discussion

For each data set under consideration, the data has been normalised and binned prior to any scaling or transformation techniques having been conducted. For ease of reference the normalised, binned spectra are referred to as "unscaled" data, and autoscaling, Pareto scaling and the glog transformation are all referred to as "scaling" methods. Furthermore, as described in the Methods section, the glog transform must initially be calibrated once for each type of biological sample. The resulting calibration parameter (Table 1) can then be used for all subsequent metabolomics analyses of that sample type that use the same NMR acquisition and processing parameters.

**Table 1:** Parameter values for all glog transformations.

Data type	Transform	λ value	*y*_0 _value
1D NMR, mussel muscle	glog	2.0025 × 10^-8^	-
	extended glog	1.2689 × 10^-8^	8.7026 × 10^-5^
pJRES NMR, dog urine	glog	2.3024 × 10^-9^	-
	extended glog	1.5175 × 10^-9^	4.9506 × 10^-5^
2D JRES NMR, fish liver	glog	6.9974 × 10^-14^	-
	extended glog	4.0877 × 10^-12^	1.575 × 10^-6^

### Data Structure

**Figure 1:**
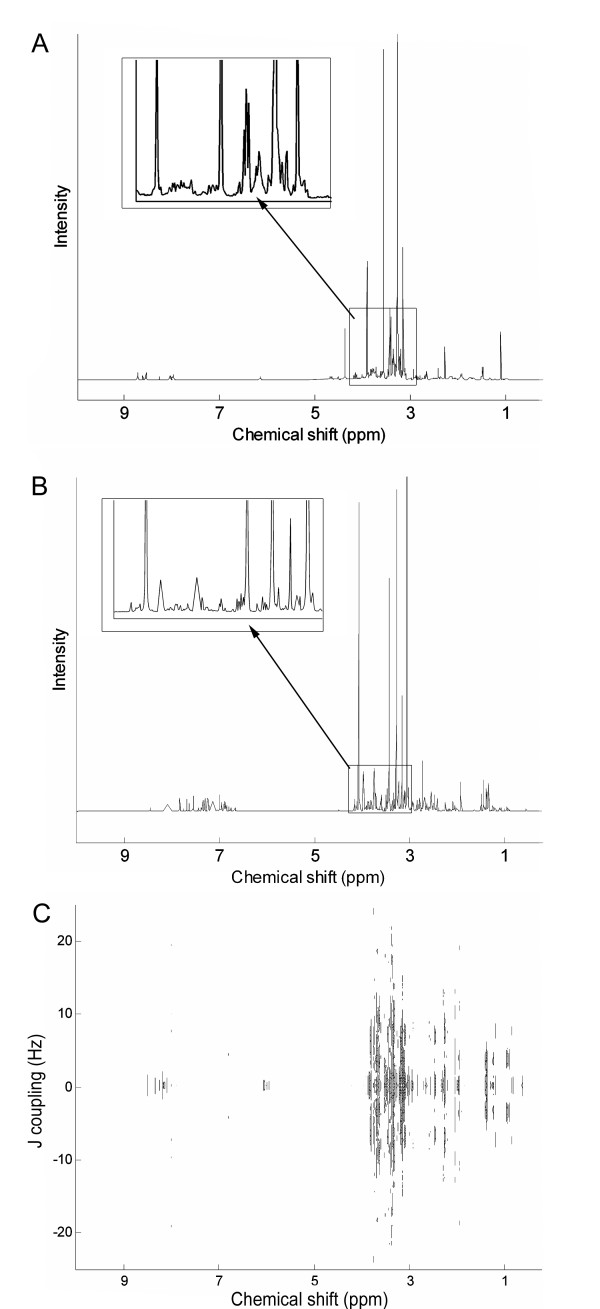
**Representative NMR spectra prior to the application of any scaling**. (A) 1D NMR spectrum of mussel adductor muscle, (B) pJRES NMR spectrum of canine urine, (C) intact 2D JRES NMR spectrum of a fish liver.

Figure 1 shows typical NMR spectra from metabolomics studies. The most commonly used experiment type is shown in Figure 1A, a 1D ^1^H NMR spectrum, which contains hundreds of peaks congested closely together that range in intensities across several orders of magnitude. This range in peak intensities becomes problematic when conducting multivariate analyses of a series of these spectra, since the technical variance (arising from sample preparation and analytical measurement) varies somewhat linearly with peak intensity (see additional file 1). This non constant variance across the NMR peaks (or bins if the NMR spectra have been binned) can then bias multivariate analyses such as PCA as the largest peaks can dominate the first few PCs due to their large (technical) variance, irrespective of the involvement of these peaks in discriminating the classes of biological samples.

Figure 1B shows the 1D skyline projection of a 2D JRES spectrum (termed pJRES), which is similar in appearance to the 1D spectrum. There is a large difference between the intensities of the smallest and largest peaks, again implying that a series of these spectra would show non constant variance. Finally, Figure 1C shows the contour plot of a typical spectrum from a 2D JRES NMR experiment. Here the peaks are less crowded than in the 1D spectra as they have been dispersed along a second dimension, and are symmetrically located about the 0 Hz line.

Prior to assessing the effects of scaling on variance, it is important to contrast the technical versus biological variability in the datasets. This can be achieved by calculating the median and range of the coefficients of variation (CV) for all the bins across a series of NMR spectra. Technical variability is measured by the CV of the technical replicates, and biological variability (which also includes technical variability) by the CV of the biological dataset. For the mussel 1D NMR data, the median CV of the technical replicates is 6.5% (range of 0.4–30.6%). In contrast, the median CV of the mussel biological data is 22.6% (range of 7.2–128.4%). Clearly the technical variance is a significant proportion of the biological variance, and therefore must be treated appropriately prior to multivariate analysis. Similar results are found for the two other data sets: the dog pJRES NMR data has median CVs of 13.4% (technical) and 52.1% (biological) with ranges of 0.6–70.4% and 14.6–272.1%, respectively. And for the fish 2D JRES NMR data the median CVs are 23.0% (technical) and 48.4% (biological) with ranges of 1.5–88.2% and 13.6–228.5%, respectively.

### Effects of Scaling on Variance

**Figure 2:**
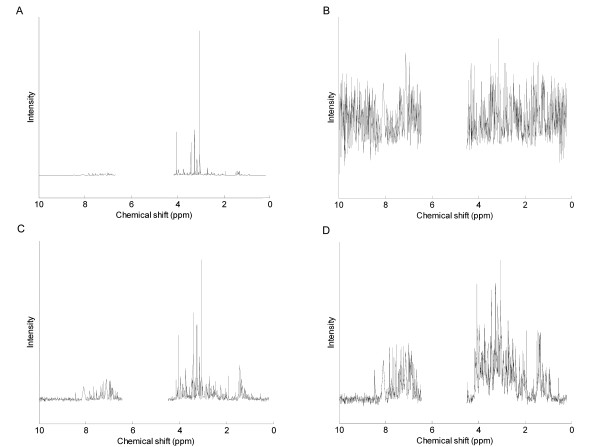
**Effects of scaling on the appearance of a pJRES canine urine NMR spectrum**. (A) Unscaled spectrum, (B) autoscaled spectrum, (C) Pareto scaled spectrum, (D) glog transformed spectrum. The region between 4.50–6.45 ppm contained the urea and residual water peaks and was therefore excluded.

Scaling techniques are applied after the other processing steps, such as normalisation and binning, have been completed and can radically change the appearance and structure of the spectra of all the different NMR data sets. For example, the canine urine data set is shown in Figure 2, where the effects of autoscaling, Pareto scaling and the glog transform are clearly visible. In particular, for both the Pareto scaled (Figure 2C) and glog transformed (Figure 2D) spectra, the weak peaks have been scaled up in intensity while the stronger peaks have been scaled down, the effect being more pronounced for the glog data set. The signal to noise ratio is particularly low for the autoscaled spectrum (Figure 2B), where it is difficult to distinguish between a peak representing a metabolite and the noise.

**Figure 3:**
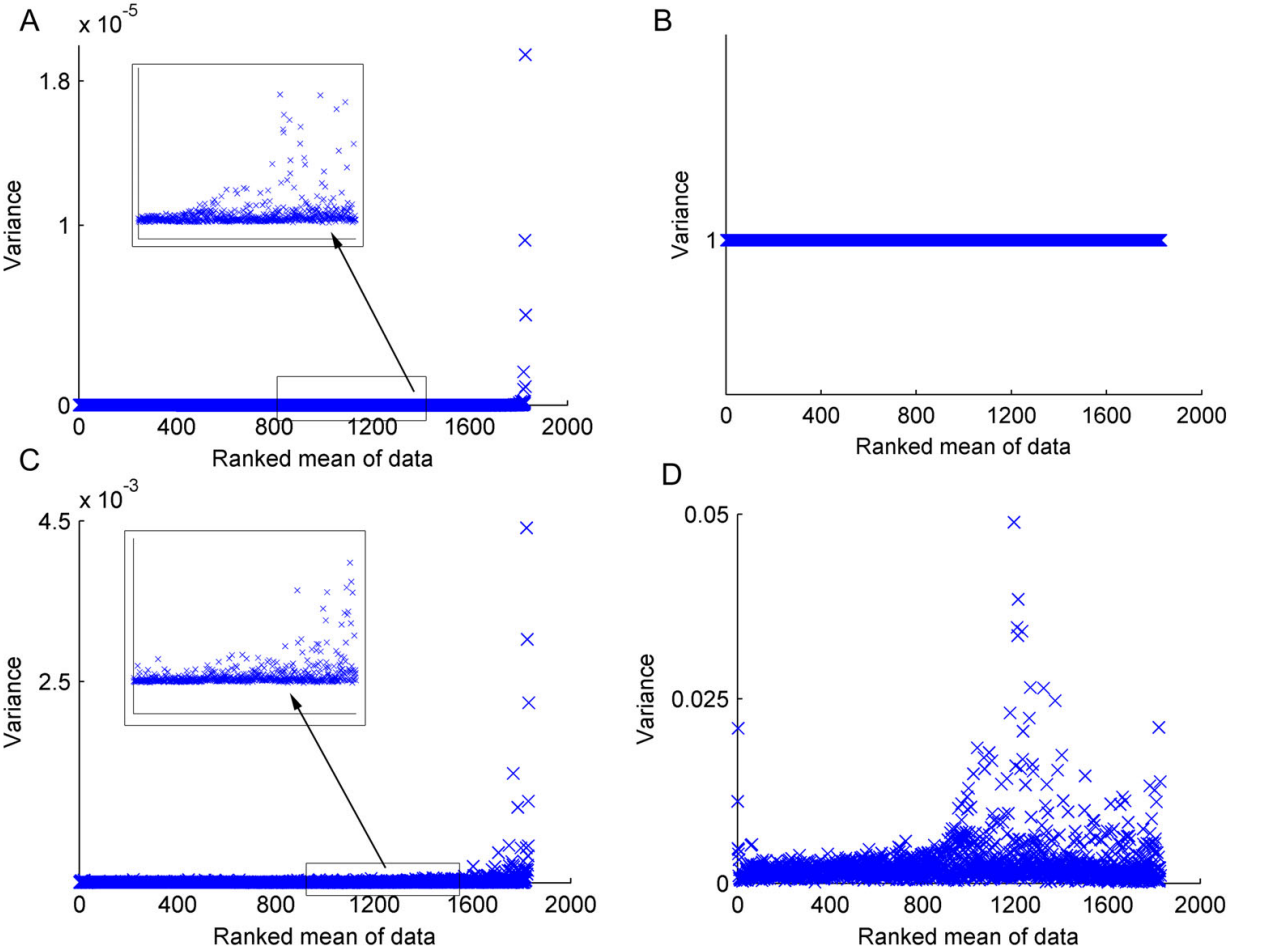
**Effects of scaling on the variance of the six technical replicates of the pooled invertebrate muscle sample**. Each plot shows the variance of every bin versus the bin number, where the bin numbers have been ranked according to their mean intensities; i.e. the highest intensity bins appear on the right of each plot. Bin variances are shown for (A) unscaled data, where the insert shows a zoomed in section, (B) autoscaled data, (C) Pareto scaled data, (D) glog transformed data.

The appearance of the spectra is only one indication of the structure of the processed data. For more information specifically relating to the ability of the scaling techniques to minimise technical variance, it is more useful to examine the variance exhibited by the bins across the spectra of technical replicates. Figure 3 shows the relationship between the variance associated with each bin and the mean value of that bin across all of the technical replicates. Here the mean intensities have been ranked such that the left of the plots describe the bins with the smallest mean values and the right hand sides show the bins with the largest mean intensities in the data set. For the unscaled data (Figure 3A), it appears that the majority of the bins have a similar variance while a few bins with large mean intensity possess a large variance. In fact on closer inspection this behaviour is repeated across the ranked bins (see insert, Figure 3A), highlighting the large range in the variance throughout the data set – even for technical replicates where the differences between spectra are minimal. Subsequently, a PCA of these unscaled spectra would result in the bins with largest variances (which correspondingly have the largest means; Figure 3A) contributing most to the first few principal components. These bins, whilst being the most intense in the data set, may not however hold the most relevant biological information.

Scaling methods can radically change the variance structure of the data set. Figure 3B shows the variance versus ranked mean for the mussel technical replicates after the spectra have been autoscaled. Here, the variance is now totally uniform, with the variance of every bin set to unity. This removes any bias that may arise due to the peak intensity. However, every bin is now treated equally, giving no differentiation between wanted signals representing peaks and unwanted signals such as noise. For Pareto scaled data (Figure 3C), there is considerable similarity in appearance to the unscaled data since there are a few bins with large mean intensity that clearly have a large variance. This structure is also repeated amongst the bins of lower mean intensity in a similar manner to the unscaled data (see insert, Figure 3C). This highlights that the Pareto scaled spectra also have a large range of variance throughout the data set, which would affect which bins contribute to the loadings in a PCA. Finally, Figure 3D shows the impact of the glog transform upon the variance of the spectra. Here the variance structure is very different from the other scaling methods as there is a wide range of bin intensities giving rise to bins with similar variances, demonstrating the variance stabilising effect of this transform. Although there are still bins with higher variances compared with the majority of bins, these bins are spread throughout the range of bin intensities and so removes any bias of PCA towards the largest peaks.

### Effects of Scaling on Classification Accuracy

**Figure 4:**
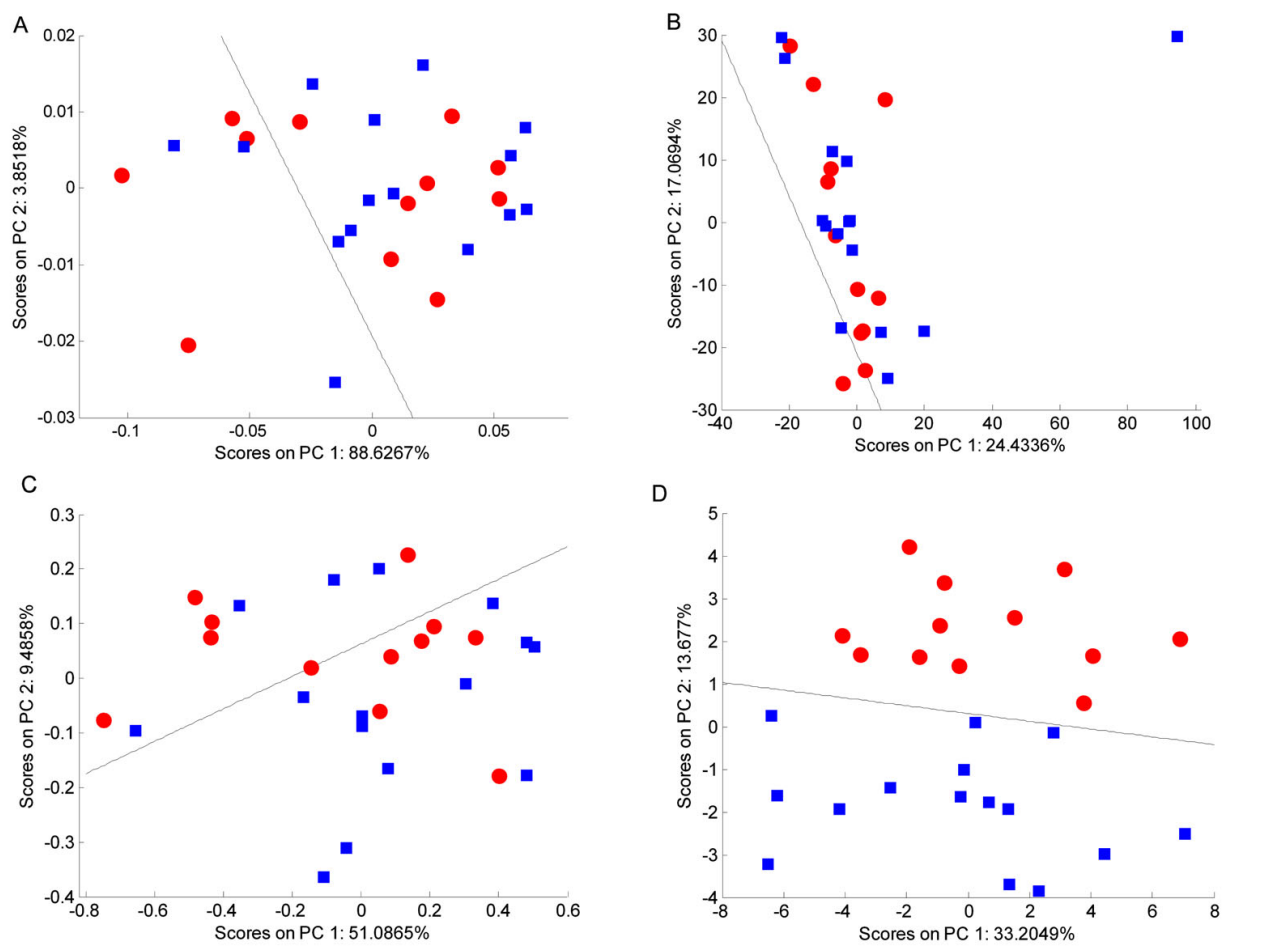
**PCA scores plots of the 1D NMR spectra of mussel adductor muscle**. (A) Unscaled data, (B) autoscaled data, (C) Pareto scaled data, (D) glog transformed data. The red circles represent the hypoxic samples whilst the blue squares represent the normoxic samples. The black line represents the decision boundary between the classes constructed using LDA.

**Figure 6:**
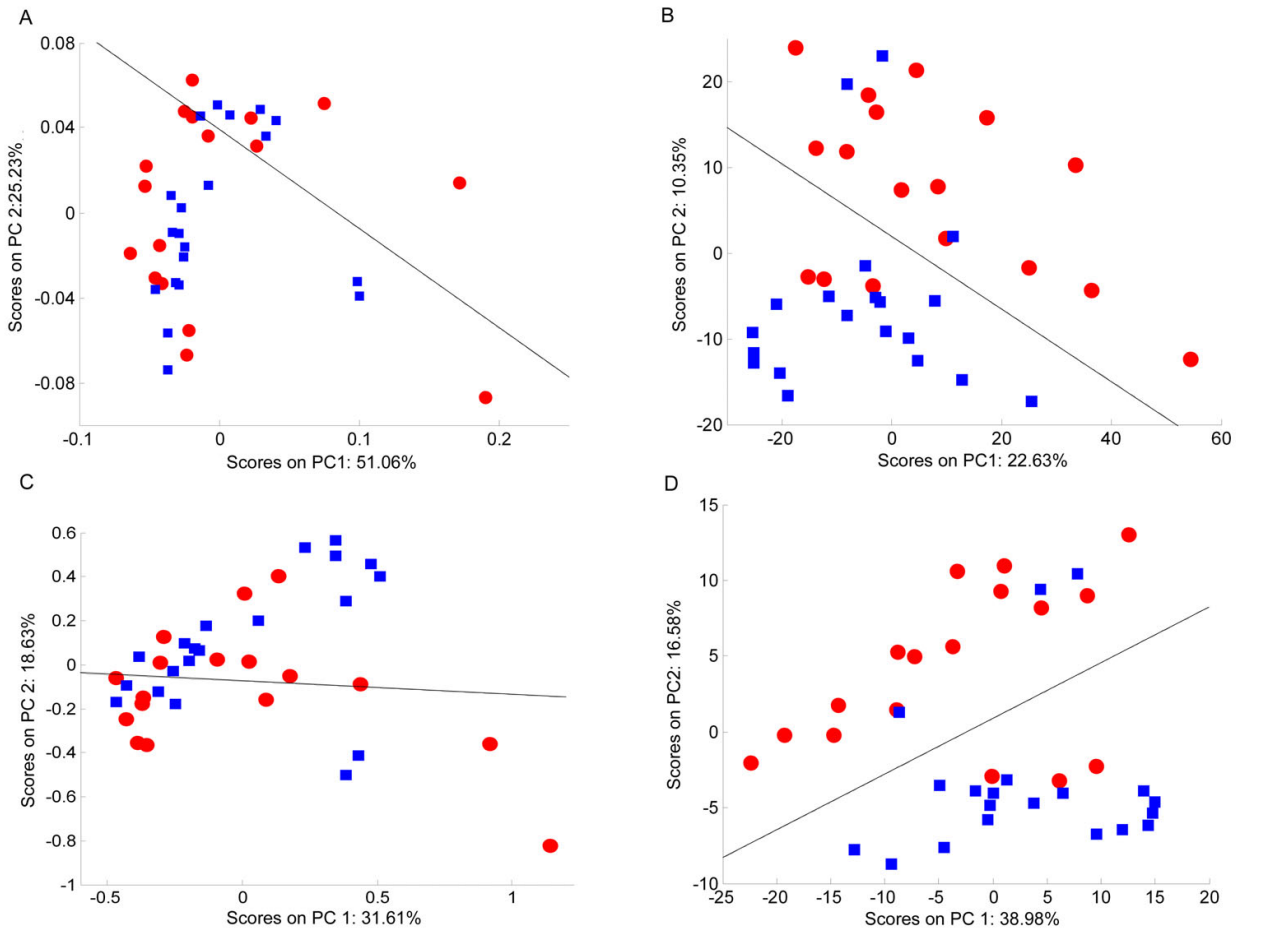
**PCA scores plots of the pJRES NMR spectra of canine urine**. (A) Unscaled data, (B) autoscaled data, (C) Pareto scaled data, (D) glog transformed data. The red circles represent the samples from Labradors, with the blue squares representing the Miniature Schnauzer samples. The black line represents the decision boundary constructed using LDA.

**Figure 8:**
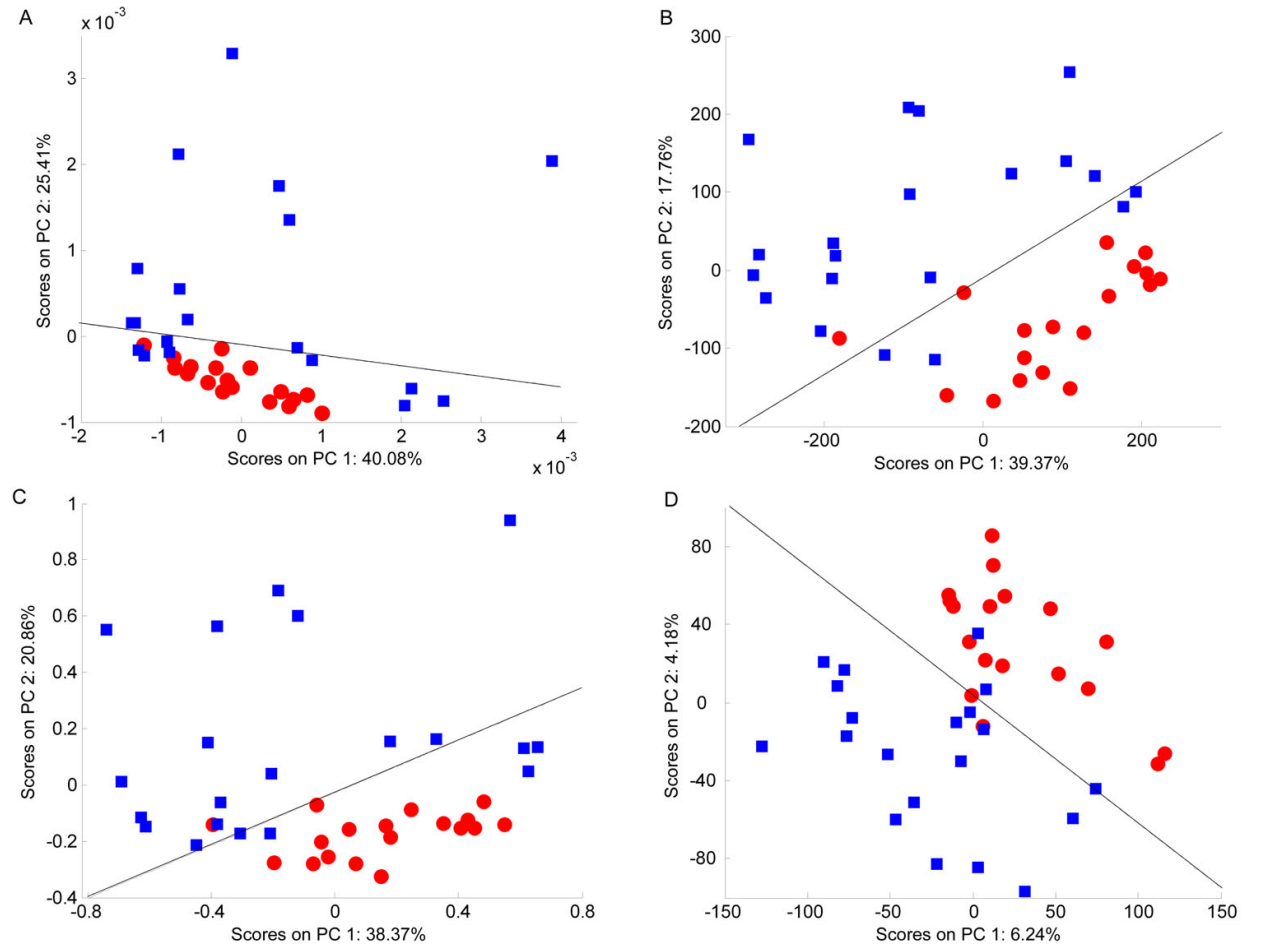
**PCA scores plots of the intact 2D JRES NMR spectra of fish liver**. (A) Unscaled data, (B) autoscaled data, (C) Pareto scaled data, (D) glog transformed data. The red circles represent fish sampled from the River Alde and the blue squares represent fish from the River Tyne. The black line represents the decision boundary between the classes constructed using LDA.

**Figure 10:**
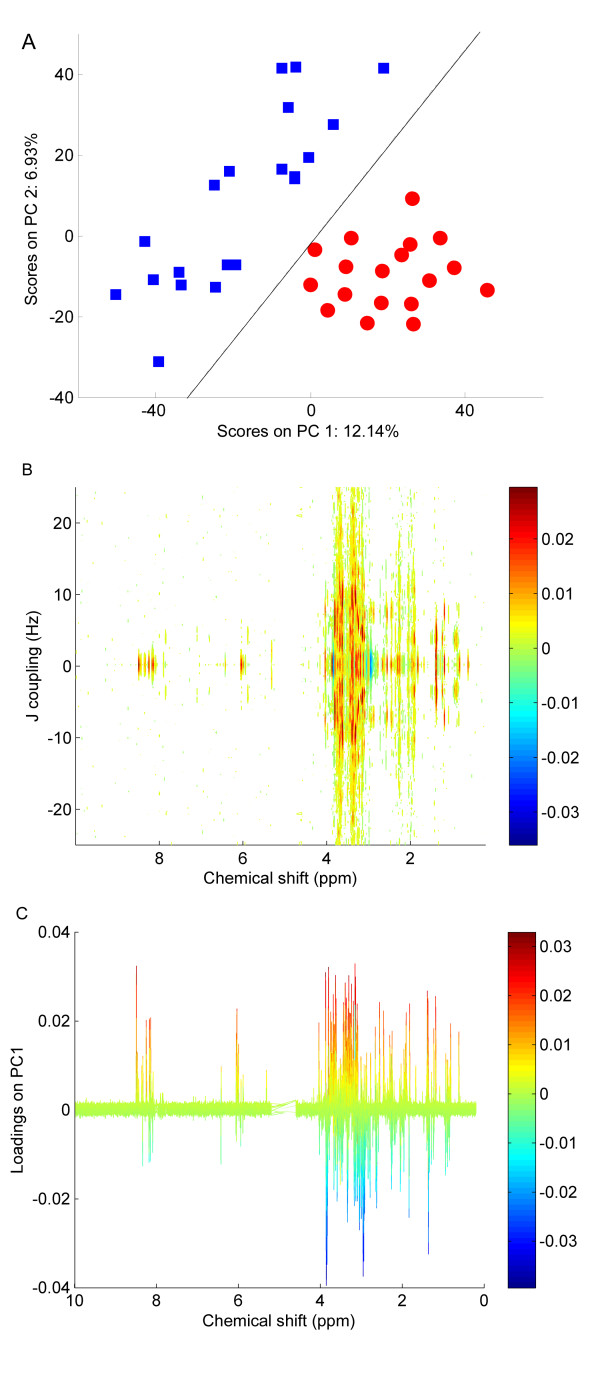
**PCA of extended glog transformed 2D JRES NMR spectra of fish liver**. (A) Scores plot where red circles represent the fish sampled from the River Alde and the blue squares represent fish from the River Tyne. The black line represents the decision boundary between the classes constructed using LDA. (B) Aerial view of the corresponding PC1 loadings plot presented in the format of a 2D JRES spectrum, with J-couplings along one axis to facilitate metabolite identification. (C) Side view of the same loadings plot as in B, highlighting the metabolites that are at higher concentration (red) in fish liver collected from the River Alde.

PCA was employed to provide an unbiased method to evaluate the usefulness of the scaling techniques, since this provides a clear strategy to observe the effects of the scaling on the variance of the data. However, all the transformations are equally applicable as a processing step prior to supervised multivariate analysis such as PLS-DA. Also, to provide a quantitative method to evaluate the models, LDA was then applied to the first and second PCs. The solid black line in Figures 4, 6, 8 and 10 represents the border between the decision regions generated by the LDA which were used to determine if a sample was correctly or incorrectly classified. Table 2 summarises the classifier statistics used to evaluate the effects of the various scaling methods on the PCA models across all three data sets.

**Table 2:** Classification statistics for each PCA model constructed.

Data type	Scaling	Sensitivity	Specificity	Correctly classified	Cross-validation accuracy
1D NMR, mussel muscle	unscaled	0.333	0.800	16 of 27	37.04%
	autoscaled	0.083	0.933	15 of 27	33.33%
	Pareto	0.500	0.733	17 of 27	51.85%
	glog	1.000	1.000	27 of 27	100.00%
	extended glog	1.000	0.86667	25 of 27	92.60%
pJRES NMR, dog urine	unscaled	0.294	0.750	20 of 37	32.43%
	autoscaled	0.824	0.850	31 of 37	83.78%
	Pareto	0.530	0.700	23 of 37	56.76%
	glog	0.824	0.850	31 of 37	83.78%
	extended glog	0.824	0.850	31 of 37	83.78%
2D JRES NMR, fish liver	unscaled	1.000	0.550	29 of 38	68.42%
	autoscaled	0.944	0.800	33 of 38	63.16%
	Pareto	0.944	0.800	33 of 38	86.84%
	glog	0.889	0.850	33 of 38	86.84%
	extended glog	1.000	1.000	38 of 38	100.00%

#### Mussel adductor muscle samples

Figure 4 shows the PCA scores plots for the models generated from the 1D NMR spectra of mussel adductor muscle. The two classes correspond to muscle obtained from normally respiring animals and from hypoxic mussels. Clearly, the unscaled data has little class-related structure, with samples of both classes intermingled with each other (Figure 4A). The decision boundary created by the LDA provides a benchmark of 16 of 27 samples correctly classified to compare against the scaled data sets (Table 2). For the autoscaled data set there is one predominant cluster of mixed samples and a single outlier (Figure 4B). Qualitatively, there is no improvement to the scores plot over the unscaled data, and the LDA decision regions show a slight decrease in accuracy, only correctly classifying 15 of the 27 samples. The Pareto scaled data appears similar to the unscaled samples, since there is no obvious discrimination between the two classes; 17 samples are correctly classified (Figure 4C). The scores plot for the glog transformed data set shows a totally different structure with complete separation of the two classes along PC2 (Figure 4D). The LDA forms a decision region which separates the two classes entirely, such that all 27 samples are correctly classified.

**Figure 5:**
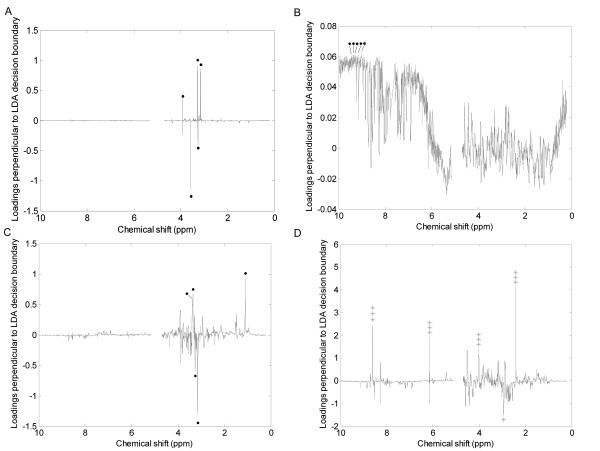
**PCA loadings plots of the 1D NMR spectra of mussel adductor muscle**. (A) Unscaled data, (B) autoscaled data, (C) Pareto scaled data, (D) glog transformed data. The plots represent the loadings perpendicular to the decision line calculated by using LDA on each of the scaled data sets. The 5 largest bins in each plot have each been tested as potential biomarkers to discriminate between the two classes. Key: (solid circle) bin is not significantly different; (*) p < 0.05; (**) p < 0.01; (***) p < 0.001.

Figure 5 shows the PCA loadings plots for each of the scaling methods applied to the mussel data set. Each plot shows the loadings generated for the PC perpendicular to the LDA decision line calculated for that scaling, so as to best describe the differences between the hypoxic and control samples. Large loadings indicate that the bin has a large contribution to the PC and hence could potentially discriminate between the two classes. Identifying which bins (and hence which metabolites) best separate the different samples is a simple way of identifying potential biomarkers and hence can be used as a second approach to evaluate the effects of each scaling method.

The 5 largest bins in each loadings plot have been tested as potential biomarkers using one-way ANOVAs. Clearly, as shown in Figures 5A, 5B and 5C for the unscaled, autoscaled and Pareto scaled data respectively, none of these bins are significantly different between the two classes and so are poor biomarkers. In contrast, only the glog transformed spectra yielded bins with the largest loadings that are all significantly different between the hypoxic and control animals (Figure 5D). This highlights a significant benefit of the glog transform for discovering useful and significant biomarkers from NMR metabolomics data.

#### Canine urine samples

For the pJRES NMR data set of urine samples from two breeds of dog, the processing methods show a similar effect upon the data (Figure 6). The PCA scores plot for the unscaled data shows little noticeable structure between the different classes, with the LDA classifier correctly identifying only 20 of the 37 samples (Figure 6A and Table 2). Pareto scaling performs only slightly better, also with no noticeable separation of the two classes, and only 23 samples correctly classified (Figure 6C). However, both the autoscaled (Figure 6B) and glog transformed (Figure 6D) data yield improved classifications of 31 of the 37 samples, with the same six samples being misclassified. The margin of separation between the two distinct clusters remains approximately the same for the autoscaled and glog transform analyses, giving no clear 'best' scaling method for this data set. The explanation behind the misclassification of the 6 samples is beyond the scope of this study, although it is important to realise that this potentially interesting result was only revealed when the data set was appropriately scaled to reduce the effects of technical variance.

**Figure 7:**
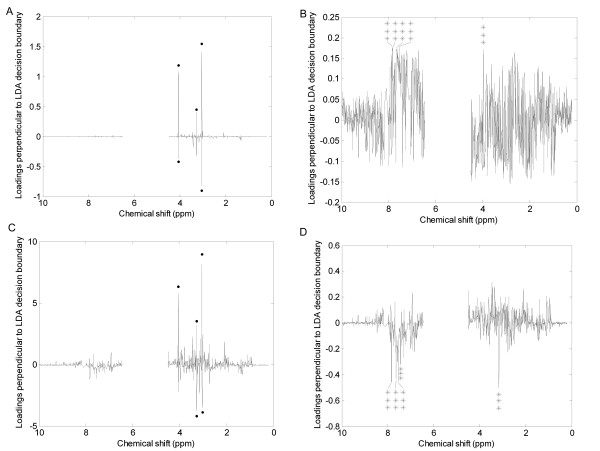
**PCA loadings plots of the pJRES NMR spectra of canine urine**. (A) Unscaled data, (B) autoscaled data, (C) Pareto scaled data, (D) glog transformed data. The plots represent the loadings perpendicular to the decision line calculated by using LDA on each of the scaled data sets. The 5 largest bins in each plot have each been tested as potential biomarkers to discriminate between the two classes. Key: (solid circle) bin is not significantly different; (*) p < 0.05; (**) p < 0.01; (***) p < 0.001.

For the unscaled and Pareto scaled data that produced the lowest classification accuracies, the loadings plots for the PC perpendicular to the LDA decision line (Figures 7A and 7C) yielded no useful biomarkers since none of the bins with the largest loadings are significantly different between classes. However, for the autoscaled and glog transformed data that produced better classification, the loadings plots perpendicular to the LDA decision line (Figures 7B and 7D) both identified bins that are significantly different between the two breeds of dog. Also, several bins were predicted as potential biomarkers by both analyses.

#### Fish liver samples

**Figure 9:**
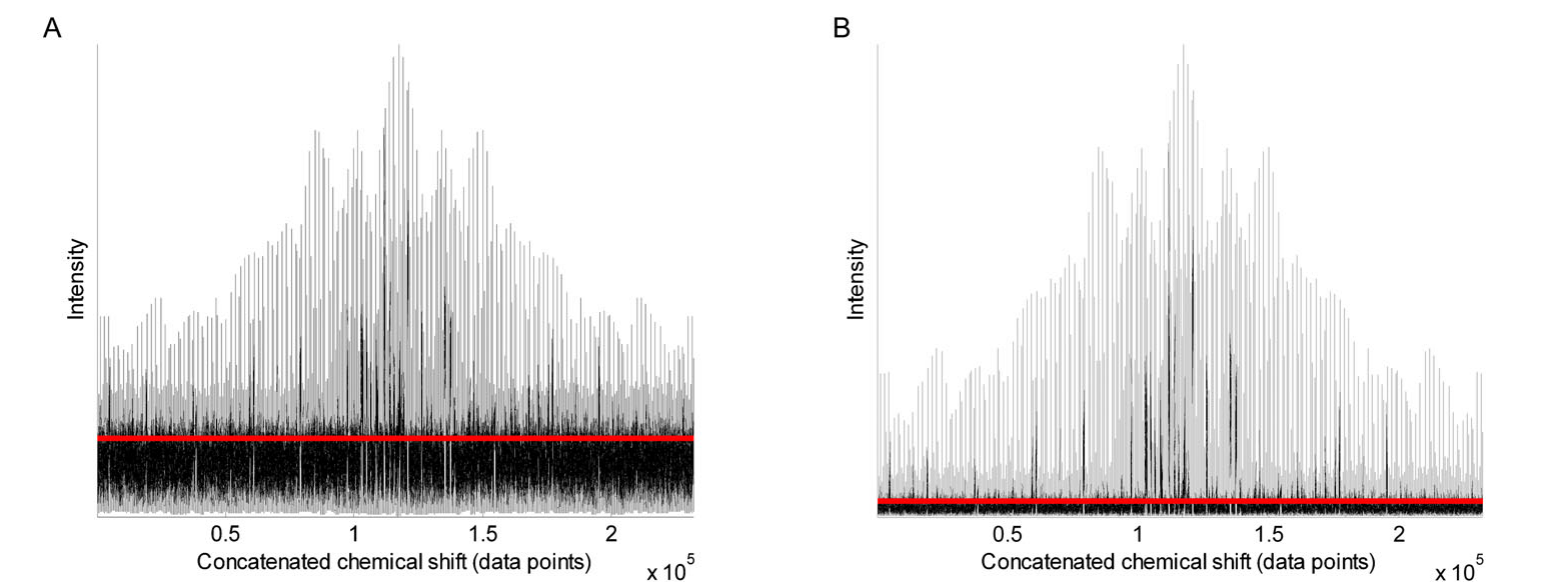
**Concatenated 2D JRES NMR spectra of fish liver**. (A) Spectra following the standard glog transformation. (B) Spectra after the extended glog transformation has been applied. Transformation parameters are listed in Table 1 and the red line indicates three times the standard deviation of the noise, regarded as the largest noise peaks.

The PCA scores plots from the analysis of the intact 2D JRES NMR data are shown in Figure 8, where the two classes correspond to fish sampled from different rivers in the UK. Here, the appearance of the four scores plots is somewhat similar for the unscaled and scaled data. In all cases there is partial separation of the two classes, and the LDA decision line reveals that 29 of 38 samples are correctly classified for the unscaled data which improves to 33 of 38 samples following autoscaling, Pareto scaling and glog transformation (Table 2). Further examination of the glog transformed data set reveals that only a small proportion of the variance is captured by the first two PCs in the PCA model. This unexpected result can be explained by examining the glog transformed data itself. Figure 9A shows a glog transformed 2D JRES spectrum following concatenation of each of the slices along the J-coupling axis into a single row vector, which facilitates a comparison of the peak heights compared to the noise. The glog transformation has not only increased the heights of the small peaks relative to the larger ones, but has also greatly magnified the noise in the spectrum.

An algorithm to increase the relative signal to noise ratio of the data was then investigated by extending the glog transformation to include an additional parameter, as shown in equation (4). Figure 9B shows the same 2D JRES spectrum after application of the extended glog transformation. The PCA scores plot of the extended glog transformed data is also changed (Figure 10A) compared to the original glog transformed data (Figure 8D), with the variance expressed by the first two PCs almost doubled to 12.1% and 6.9%. The most noteworthy result is that the reduction of noise using the extended glog transform also improves the LDA classifier, with all 37 of 37 samples now correctly assigned to their correct classes and separation between the two classes in PCA space now readily apparent (Figure 10A).

The corresponding PC1 loadings plot for the scores plot in Figure 10A is shown in two different orientations in Figures 10B (top view) and 10C (side view). When used in combination with the extended glog transformation the resulting loadings plot provides a powerful visualisation tool from which the metabolic differences between the two sample classes can be identified. In particular, the significant advantages over the more traditional 1D loadings plots derived from both 1D NMR data as well as 1D pJRES data [15] include the decreased congestion of peaks and the preservation of J-coupling information. Ultimately this approach could increase the confidence of metabolite identification in NMR metabolomics.

## Conclusion

We have demonstrated that autoscaling, Pareto scaling and the glog and extended glog transformations can significantly alter the variance structure of NMR metabolomics data, which in turn can improve the classification accuracy of multivariate models generated from the scaled data. This can help to extract important information from data sets, since improving the discrimination between sample classes can help to identify metabolic biomarkers. Specifically, we have demonstrated that the glog and extended glog transformations achieve the best, or equal best, classification accuracy compared to unscaled, autoscaled and Pareto scaled data on three example data sets. A classification accuracy of 100% was achieved for two data sets – the effect of hypoxia in invertebrate muscle extracts and the effect of sampling location on fish liver extracts – and an accuracy of 31 of 37 correctly classified for a third dataset examining breed discrimination using dog urine. Furthermore, from an analysis of the top five peaks in each of the corresponding PCA loadings plots, we have confirmed that glog transformed data is considerably better at discovering metabolic biomarkers that can discriminate significantly between sample classes. We have also confirmed the broad applicability of the glog approach using three disparate data sets from different biological samples using 1D NMR spectra, 1D projections of 2D JRES spectra, and intact 2D JRES spectra. Finally, we have reported an extension to the original glog algorithm that effectively suppresses the noise, which was critical for the analysis of intact 2D JRES spectra. In conclusion, we have thoroughly evaluated and proven the benefits of utilising the glog transformation for stabilising the technical variance associated with metabolomics experiments, which can lead to significantly beneficial effects on the discrimination between sample classes using multivariate analysis.

## Methods

Three data sets were used to highlight the broad applicability of the generalised log transformation across multiple biological species and sample types. The three data sets comprised spectra of mammalian (canine) urine, extracts of marine mussel adductor muscle, and extracts of fish liver. The preparation, NMR analysis and processing of each is described below.

### Sample Preparation and Collection of NMR Spectra

#### Canine urine

Free-catch urine samples were collected over several days from two breeds of dog (17 samples from three male Labradors and 20 samples from four male Miniature Schnauzers), frozen at -80°C, and subsequently prepared and analysed using the methods described elsewhere [22]. Briefly, urine was diluted in a sodium phosphate buffer (pH 7.0; 100 mM final concentration) containing sodium 3-trimethylsilyl-2,2,3,3-d4-propionate (TMSP; 1 mM final concentration), 0.2% sodium azide and 8% D_2_O. The sample pH was then manually adjusted to 7.05 (± 0.05) using 1 M HCl or NaOH. Samples were analyzed on a Bruker 500 MHz NMR spectrometer equipped with a 5 mm cryoprobe and BACS-60 automatic sample changer. 2-D ^1^H, ^1^H JRES NMR spectra were collected with 16 increments using excitation sculpting to suppress the water resonance, and transients were processed using methods described previously [15], yielding 1-D skyline projections of the JRES spectra (termed pJRES). In addition to the 37 individual urine samples, an additional pooled sample was split into 5 fractions and each of these was then prepared and analysed separately, using the same methods as above. This provided the spectra of technical replicates needed to calibrate the generalised log transform.

#### Mussel adductor muscle

Muscle tissues were dissected from two groups of Mediterranean mussels (*Mytilus galloprovincialis*), the first group being hypoxic (i.e. oxygen deficient; n = 12) and the second group normoxic (n = 15). The tissues were prepared using a methanol:chloroform extraction protocol as recently reported [23]. Polar extracts were dried and then resuspended in 100 mM sodium phosphate buffer (pH 7.0; 1 mM TMSP; 10% D_2_O). 1-D ^1^H NMR spectra of the polar metabolites were collected, as described previously [24]. Similar to the canine urine study, an additional pooled tissue sample was homogenised, split into 6 fractions and then each fraction was extracted and analysed separately, providing spectra of technical replicates.

#### Fish liver

European flounder (*Platichthys flesus*) were sampled from the River Alde, UK (n = 20) and the River Tyne, UK (n = 18). Liver tissue was rapidly dissected and then extracted using the methanol:chloroform protocol as above [23]. All polar extracts were dried and resuspended in 90% H_2_O and 10% D_2_O with sodium phosphate buffer (100 mM; pH 7.0) containing 0.5 mM TMSP. 2-D ^1^H, ^1^H JRES NMR spectra were collected using methods described above. Again, an additional pooled tissue sample was homogenised, split into 5 fractions and then each was extracted and analysed separately, providing spectra of technical replicates.

#### Technical Replicates

It should be noted that for all data sets, the technical replicates form an integral part of calibrating the glog transformation. A minimum of five or six replicates should be generated from a single homogenous pool of the relevant biological material for each data set. Ideally, this pool of biological material is formed by mixing several smaller amounts of different samples from all experimental classes (e.g., control and stressed).

### Data Processing

The 1-D, pJRES and 2D JRES NMR spectra were converted to an appropriate format for multivariate analysis using custom-written *ProMetab *software [15] running within MATLAB (version 7.1; The MathWorks, Natick, MA). All spectra were sectioned into 1960 chemical shift bins between 0.2 and 10.0 ppm, corresponding to a bin width of 0.005 ppm. Note that the 2D JRES spectra were not "binned" along the J coupling dimension at this stage of the processing. Next, a series of bins were removed from each data set: for the canine urine from 4.50–6.45 ppm (residual water and urea); for the mussel adductor muscle from 4.70–5.15 ppm (residual water) and 7.60–7.76 ppm (chloroform); and for fish liver from 4.60–5.20 ppm (residual water). The spectra for each data set were then normalised to a total spectral area of unity for ease of comparison between samples. Next, due to slight pH-induced chemical shift variations of some peaks between samples, groups of bins were each compressed into single bins: for the canine urine ten regions were compressed between 2.40–2.425, 2.52–2.57, 2.66–2.71, 2.935–2.955, 2.96–2.98, 3.105–3.130, 3.72–3.77, 3.955–3.990, 7.08–7.20 and 8.00–8.18 ppm; for the mussel adductor muscle between 7.08–7.10 and 7.84–7.875 ppm; and for fish liver five regions were compressed between 7.74–7.77, 7.77–7.79, 7.94–7.955, 7.97–8.03 and 8.23–8.25 ppm. Compression regions were chosen by visually inspecting the superimposed NMR spectra and then selecting regions of the spectra that showed pH or matrix induced chemical shift variation. Finally, for the fish liver only, the increments of each intact 2D JRES spectrum (i.e. the rows of the 2D data matrix representing each spectrum) were concatenated into a single row vector of dimension 232,448 containing the intensities of each bin in the spectrum, allowing the JRES spectra to be analysed in a similar manner to the 1D and pJRES spectra, described below.

### Scaling Methods

After each data set was binned, normalised and bin compressed – and for the intact 2D JRES spectra, concatenated – the following scaling techniques were applied:

#### Autoscaling

The variance of each bin was scaled to unity by dividing the intensity of each bin by the standard deviation of that bin; note that mean centring was not applied yet.

#### Pareto scaling

The intensity of each bin was divided by the square root of the standard deviation of that bin; again, mean centring was not applied at this point.

#### Glog transformation

**Figure 11:**
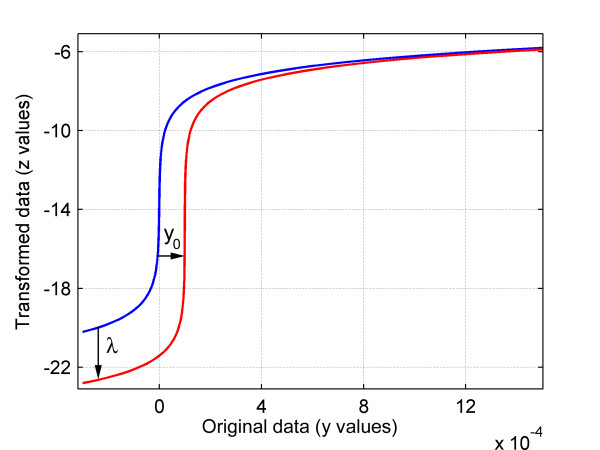
**Plot of the generalised logarithm and extended generalised logarithm functions**. The glog was plotted using a λ value of 1 × 10^-12 ^(solid blue line) and the extended glog was plotted using a λ value of 1 × 10^-13 ^and a *y*_0 _value of 1 × 10^-4 ^(solid red line). The effects of changing the two transformation parameters are indicated on the diagram by the black arrows. Since the transformed intensities include negative values, following the transformation each spectrum is linearly shifted upwards so that each baseline is located at zero intensity.

The glog transformation is given in equation (1), where *z *is the intensity of the transformed data, *y *is the intensity of each original bin, and λ is a parameter that affects the gradient of the function (see Figure 11). This parameter must be found prior to using the transformation as it is specific to each type of biological sample and set of NMR conditions.

In order to calculate the transform that minimises the technical variance, λ is calibrated using technical replicates generated from a single pooled biological sample. The replicate spectra are processed in exactly the same manner as the biological data set, i.e. normalisation, compression regions etc, to ensure all technical variance is accounted for when calibrating the glog parameters. The calibration was achieved using a maximum likelihood method proposed by Rocke and Durbin [25]. To avoid scaling artefacts arising from the change in scale between the untransformed and transformed variables the Jacobian, of the glog function is used as a scaling factor as described by Rocke and Durbin. Here however, we have used an alternative scaling function which maintains most of the properties of the Jacobian but is computationally more robust; shown in equation (2), where *z*_*i *_represents bin *i *and *n *is the total number of bins contained within the spectrum being scaled.

J=exp⁡(∑i=1n(ln⁡zi2+λ)n)
 MathType@MTEF@5@5@+=feaafiart1ev1aaatCvAUfKttLearuWrP9MDH5MBPbIqV92AaeXatLxBI9gBaebbnrfifHhDYfgasaacH8akY=wiFfYdH8Gipec8Eeeu0xXdbba9frFj0=OqFfea0dXdd9vqai=hGuQ8kuc9pgc9s8qqaq=dirpe0xb9q8qiLsFr0=vr0=vr0dc8meaabaqaciaacaGaaeqabaqabeGadaaakeaacqWGkbGscqGH9aqpcyGGLbqzcqGG4baEcqGGWbaCdaqadaqaamaalaaabaWaaabCaeaadaqadaqaaiGbcYgaSjabc6gaUnaakaaabaGaemOEaO3aa0baaSqaaiabdMgaPbqaaiabikdaYaaakiabgUcaRGGaciab=T7aSbWcbeaaaOGaayjkaiaawMcaaaWcbaGaemyAaKMaeyypa0JaeGymaedabaGaemOBa4ganiabggHiLdaakeaacqWGUbGBaaaacaGLOaGaayzkaaaaaa@4815@

The parameter λ was optimised by minimising the variance, S, (3) over *k *technical replicates and all *n *bins in the Jacobian-scaled data vectors *w*_*j *_= *z*_*j*_*J*, giving a measure of all variance contained within the technical replicates.

S(λ)=∑j=1k∑i=1n(wij−w^i)2.
 MathType@MTEF@5@5@+=feaafiart1ev1aaatCvAUfKttLearuWrP9MDH5MBPbIqV92AaeXatLxBI9gBaebbnrfifHhDYfgasaacH8akY=wiFfYdH8Gipec8Eeeu0xXdbba9frFj0=OqFfea0dXdd9vqai=hGuQ8kuc9pgc9s8qqaq=dirpe0xb9q8qiLsFr0=vr0=vr0dc8meaabaqaciaacaGaaeqabaqabeGadaaakeaacqWGtbWucqGGOaakiiGacqWF7oaBcqGGPaqkcqGH9aqpdaaeWbqaamaaqahabaGaeiikaGIaem4DaC3aaSbaaSqaaiabdMgaPjabdQgaQbqabaGccqGHsislcuWG3bWDgaqcamaaBaaaleaacqWGPbqAaeqaaOGaeiykaKYaaWbaaSqabeaacqaIYaGmaaaabaGaemyAaKMaeyypa0JaeGymaedabaGaemOBa4ganiabggHiLdaaleaacqWGQbGAcqGH9aqpcqaIXaqmaeaacqWGRbWAa0GaeyyeIuoakiabc6caUaaa@4C58@

Here, w^
 MathType@MTEF@5@5@+=feaafiart1ev1aaatCvAUfKttLearuWrP9MDH5MBPbIqV92AaeXatLxBI9gBaebbnrfifHhDYfgasaacH8akY=wiFfYdH8Gipec8Eeeu0xXdbba9frFj0=OqFfea0dXdd9vqai=hGuQ8kuc9pgc9s8qqaq=dirpe0xb9q8qiLsFr0=vr0=vr0dc8meaabaqaciaacaGaaeqabaqabeGadaaakeaacuWG3bWDgaqcaaaa@2E33@ is calculated as the mean spectrum of all scaled and transformed technical replicates, *w*_*j*_. Minimising the variance S thus gives an optimal value for λ.

The optimisation of λ is achieved via the Nelder-Mead unconstrained non-linear minimization routine in the MATLAB optimisation toolbox. The optimised λ value was then used to transform the binned intensities of each spectrum in the full biological data set. The MATLAB code developed here is included as additional file 2.

The extended glog is given in equation (4) where an extra transformation parameter *y*_0 _has been added. As illustrated in Figure 11, *y*_0 _shifts the transformation function so that the bins with the lowest intensities are scaled by the section of the glog function which has a relatively small slope.

z=ln⁡((y−y0)+(y−y0)2+λ)
 MathType@MTEF@5@5@+=feaafiart1ev1aaatCvAUfKttLearuWrP9MDH5MBPbIqV92AaeXatLxBI9gBaebbnrfifHhDYfgasaacH8akY=wiFfYdH8Gipec8Eeeu0xXdbba9frFj0=OqFfea0dXdd9vqai=hGuQ8kuc9pgc9s8qqaq=dirpe0xb9q8qiLsFr0=vr0=vr0dc8meaabaqaciaacaGaaeqabaqabeGadaaakeaacqWG6bGEcqGH9aqpcyGGSbaBcqGGUbGBcqGGOaakcqGGOaakcqWG5bqEcqGHsislcqWG5bqEdaWgaaWcbaGaeGimaadabeaakiabcMcaPiabgUcaRmaakaaabaGaeiikaGIaemyEaKNaeyOeI0IaemyEaK3aaSbaaSqaaiabicdaWaqabaGccqGGPaqkdaahaaWcbeqaaiabikdaYaaakiabgUcaRGGaciab=T7aSbWcbeaakiabcMcaPaaa@45E6@

The parameter *y*_0 _was calibrated by first estimating the noise contained within the spectra of technical replicates. The noise level was set to the smallest standard deviation of 32 equally sized regions across the spectra [26]. The shift *y*_0 _of the glog function was then determined by calculating the point in glog where the slope of the function increases, i.e. by calculating the point where the second derivative of z in equation (5) has its maximal value. This point was typically set to three times the noise value of the spectrum. This shift ensures that the noise of the spectrum is minimally scaled by the flat region of the glog function while the larger intensity bins remain transformed to the higher values. Thus the noise is effectively suppressed relative to those bins corresponding to low and medium intensity peaks.

d2zdy2=−y(y2+λ)−32
 MathType@MTEF@5@5@+=feaafiart1ev1aaatCvAUfKttLearuWrP9MDH5MBPbIqV92AaeXatLxBI9gBaebbnrfifHhDYfgasaacH8akY=wiFfYdH8Gipec8Eeeu0xXdbba9frFj0=OqFfea0dXdd9vqai=hGuQ8kuc9pgc9s8qqaq=dirpe0xb9q8qiLsFr0=vr0=vr0dc8meaabaqaciaacaGaaeqabaqabeGadaaakeaadaWcaaqaaiabdsgaKnaaCaaaleqabaGaeGOmaidaaOGaemOEaOhabaGaemizaqMaemyEaK3aaWbaaSqabeaacqaIYaGmaaaaaOGaeyypa0JaeyOeI0IaemyEaKNaeiikaGIaemyEaK3aaWbaaSqabeaacqaIYaGmaaGccqGHRaWkiiGacqWF7oaBcqGGPaqkdaahaaWcbeqaamaalmaameaacqGHsislcqaIZaWmaeaacqaIYaGmaaaaaaaa@4228@

Since *y*_0 _depends on the choice of λ the optimisation of λ must be carried out first followed by the calculation of *y*_0_. In some cases it may be necessary to optimise λ a second time after *y*_0 _has been set, in particular for very noisy data.

For both calibration methods described here, the minimisation routine was terminated when the absolute change in λ was less than a predetermined value (here 1 × 10^-16^) or a maximum number of iterations was completed (here 1 × 10^3^). Table 1 contains the optimised λ and *y*_0 _values for the glog and extended glog transformations for each of the three biological samples investigated.

### Analysis of Models

Each unscaled or scaled data set was then mean centred and PCA performed using PLS_Toolbox (Eigenvector Research, Inc., Wenatchee, WA, USA). Next, using the Discriminant Analysis Toolbox (Michael Kiefte, Dalhousie University, Canada [27]) Fisher's LDA was applied to the first and second PCs of the PCA scores plot, producing a decision region for each two-class problem. This decision region was then used to construct classification statistics (sensitivities and specificities) to evaluate the effects of the scaling techniques upon each data set (Table 2). Leave-one-out cross-validation was performed on the PCA-LDA models to assess the robustness of the analyses (Table 2). The coefficient of variation (CV) for each bin, given as the standard deviation divided by the mean, was calculated for each set of technical replicates, excluding bins with an intensity lower than the estimated noise level of the spectrum (i.e., the CV was calculated using only those bins that contained peaks). The median and range of these CVs were calculated for each of the three data sets. Additionally, PCA loadings plots for the 1D and pJRES data (Figures 5 and 7) were produced by constructing the linear combination of the loadings along PC1 and PC2 that is perpendicular to the LDA decision line. The loadings plot for the 2D JRES experiment, shown in 2D matrix format to mimic an intact 2D JRES spectrum (Figures 10B and 10C), was reconstructed from the row vector containing the loadings of the concatenated spectra. To evaluate the discriminatory potential of metabolic biomarkers discovered in the loadings plots, one-way analysis of variance (ANOVA) was performed on each of the 5 bins with the largest absolute loadings values, for each data set and method of scaling.

## List of abbreviations used

NMR: nuclear magnetic resonance

PCA: principal component analysis

PLS-DA: partial least squares discriminant analysis

PC: principal component

LDA: linear discriminant analysis

glog: generalised logarithm transformation

1D: one dimensional

2D: two dimensional

JRES spectrum: 2D J-resolved NMR spectrum

pJRES: 1D skyline projection of a 2D JRES spectrum

ANOVA: analysis of variance

CV: coefficient of variance

## Authors' contributions

HMP wrote the code implementing the methodology and completed the comparisons of the different scaling methods. MRV conceived of the study, participated in its completion and helped to draft the manuscript. CL and ULG conceived and tested the extended glog transformation. All authors read and approved the final manuscript.

## Supplementary Material

Additional file 1 Bin variance versus bin intensity of the technical replicates for (A) 1D mussel data; (B) pJRES dog data; (C) JRES fish data. Some low intensity bins (predominantly noise) can be seen to the left of the plots which exhibit similar variance levels; however a more linear relationship can be seen in the medium and high intensity bins. Click here for file

Additional file 2 Optimisation code. Click here for file
