# Supplementary figures and images for: Improved classification accuracy in 1- and 2-dimensional NMR metabolomics data using the variance stabilising generalised logarithm transformation
Source: BMC Bioinformatics. 2007 Jul 2;8:234. doi: 10.1186/1471-2105-8-234 (PMC1965488; doi:10.1186/1471-2105-8-234)

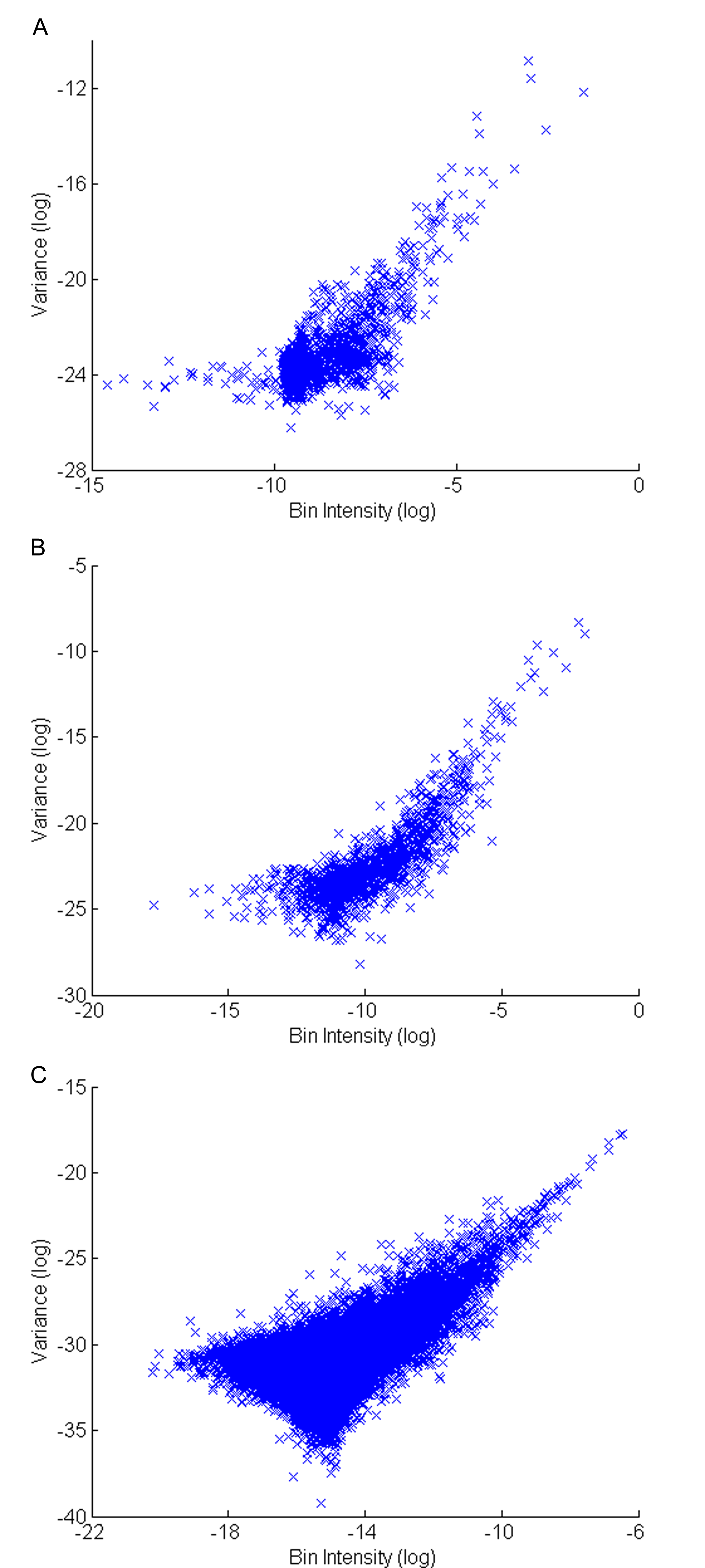

Supplement: Additional file 1 — Bin variance versus bin intensity of the technical replicates for (A) 1D mussel data; (B) pJRES dog data; (C) JRES fish data. Some low intensity bins (predominantly noise) can be seen to the left of the plots which exhibit similar variance levels; however a more linear relationship can be seen in the medium and high intensity bins. [file 1471-2105-8-234-S1.png]
