# Supplementary material for: Improved classification accuracy in 1- and 2-dimensional NMR metabolomics data using the variance stabilising generalised logarithm transformation
Source: BMC Bioinformatics. 2007 Jul 2;8:234. doi: 10.1186/1471-2105-8-234 (PMC1965488; doi:10.1186/1471-2105-8-234)
Supplement: Additional file 2 — Optimisation code. [file 1471-2105-8-234-S2.zip › Glog parameter optimisation readme.rtf]

Glog parameter optimisation readme.

Four files are contained in this .zip file
Glog parameter optimisation readme.rtf
optimise_glog_parameter.m
glog_opt.m
meanplot.m

The three .m files should be placed in your MATLAB directory. Also note that the optimisation routines both require the function fminsearch from the MATLAB optimisation toolbox (not included).
Note that this code has also been written to complement the ProMetab software (Viant, BBRC 2003, 310:943-948), but is also useable without.


Standard glog transform

To optimise ë, type at the command line

optimise_glog_parameter(x);

where x is the NxM matrix containing the N technical replicates each of length M. If using ProMetab for analysis, for example, type

optimise_glog_parameter(spec_array_bin); 

Several plots will be generated during the optimisation process
1)	A plot of the 'raw' (untransformed) variables
2)	A plot describing the variance against intensity of the raw variables
3)	A plot showing the minimisation of the SSE (for each starting point)
4)	A plot describing the variance against intensity of the transformed variables
5)	A plot of the transformed variables
At the command line, the trial values of ë and the resultant SSE are printed until a final, optimal ë value is reached. The optimised ë is then printed, along with successful starting point and the time taken for the algorithm to converge.


Extended glog transform

This is performed in a similar manner to above, but using the command

glog_opt(x)

Here, y0 will also be optimised and printed at the command line along with the optimised ë value.
